# Enhanced catalytic activity of N-heterocyclic carbene stabilized surface adatoms for CO reduction reaction

**DOI:** 10.1038/s42004-023-01066-2

**Published:** 2023-12-11

**Authors:** Yuxiang Gao, Lei Tao, Yu-Yang Zhang, Shixuan Du

**Affiliations:** 1 Institute of Physics and University of Chinese Academy of Sciences, Chinese Academy of Sciences, Beijing, 100190 China; 2 Beijing National Center for Condensed Matter Physics, Beijing, 100190 China; 3 Songshan Lake Materials Laboratory, Dongguan, 523808 China

**Keywords:** Electrocatalysis, Carbon capture and storage, Theory and computation, Computational chemistry, Surface chemistry

## Abstract

Adatom engineering represents a highly promising opportunity for enhancing electrochemical CO reduction reaction (CORR). However, the aggregation of adatoms under typical reaction conditions often leads to a decline in catalyst activity. Recent studies have revealed that N-heterocyclic carbene (NHC) can stabilize surface adatoms. Herein, based on density functional theory calculations, we reveal a significant enhancement in the catalytic activity of Cu adatoms decorated with NHC molecules for CORR. The NHC decoration strengthens the interaction between the *d*_*xy*_ orbital of the Cu adatom and the *p*_*x*_ orbital of the C atom, reducing the energy barriers in both CO hydrogenation and C-C coupling steps. Moreover, the CORR catalytic activity of the NHC decorated adatom can be further improved by tuning the side groups of NHC molecules. These results provide insights for the design of efficient CORR catalysts and offer a theoretical framework that can be extended to other hydrogenation reactions.

## Abstract (web summary)

Single atom catalysts dispersed on a surface demonstrate great promise for a variety of catalytic reactions, but their aggregation leads to a degradation of catalytic activity. Here, the authors use quantum mechanical calculations to study the catalytic activity of Cu adatoms stabilized with N-heterocyclic carbenes (NHCs) on a Cu(100) surface, finding that NHC-decoration significantly reduces the energy barriers to electrocatalytic CO hydrogenation and C–C coupling.

## Introduction

Efficient strategies for CO_2_ recycling play a crucial role in maintaining the carbon cycle and preventing the negative impact of CO_2_ emissions on global warming^1–5^. In the industry, various pathways can be utilized for CO_2_ recycling, including thermal hydrogenation, photocatalytic CO_2_ reduction, and electrochemical CO_2_ reduction. Among them, the electrochemical CO_2_ reduction reaction (CO_2_RR) is promising for converting CO_2_ into value chemicals and fuels on a practical scale^1,6–9^. Significant progress has been achieved in the two-electron reductions of CO_2_ to CO^10^. However, further reduction of CO to multiple-carbon products still suffers from poor energy efficiency and selectivity^11^. The electrocatalysts can reduce the overpotential of CO reduction reaction (CORR), accelerate the reaction rate, and improve the selectivity of value-added multiple-carbon products. Therefore, developing highly efficient electrocatalysts to produce economically valuable C_2_ products is essential for CORR^12–14^.

Several strategies have been developed to enhance the performance of electrocatalyst, including selective facet exposure^15,16^, interface engineering^17^, and surface adatom modification^18–20^. Among these strategies, the adatoms show advantages of unique electronic structures, low coordination metal atoms, strong metal-carrier interactions, and maximum atomic utilization efficiency^21,22^, which can enhance the catalytic activity and reaction selectivity^20,21,23^. For instance, surface adatom modification has demonstrated its ability to activate the catalytically inert surface of MoS_2_ for CO_2_RR, resulting in a significant improvement in its catalytic activity^24^. However, the aggregation of surface adatoms due to the large surface energy leads to the degradation of catalyst activity^25^. The key challenge is to stabilize the isolated surface adatom during CORR reaction process^26,27^. In recent years, it has been reported that N-heterocyclic carbene (NHC) molecules can stabilize surface adatoms when combined with transition metal atoms^28–31^. The NHC-decorated adatoms demonstrate stability at the temperature of 70 °C^28^, indicating that the catalysts based on the NHC-decorated adatoms are thermodynamically stable under the reaction condition^32^. Moreover, NHC molecules also possess tunable side groups that facilitate various functionalities in the fields of catalysis and materials chemistry on material surfaces and nanoparticles^33–36^.

In this paper, we propose a strategy to improve the catalytic activity of the Cu adatoms on Cu(100) surface for CORR through NHC molecules decoration. Employing density functional theory (DFT) calculations, we found that the decoration of NHC molecules enhances the interaction between the *d*_*xy*_ orbital of Cu adatoms and the *p*_*x*_ orbital of C atom in CO. As a result, the energy barriers for CO hydrogenation and C-C coupling reaction are significantly reduced. The energy barrier for CO reduction to CHO decreases from 0.970 eV to 0.440 eV, while the CO-CHO coupling reaction changes from an endothermic reaction to an exothermic one with an energy barrier of 0.452 eV. The calculation results demonstrate that the NHC decorated Cu adatoms serve as new CO adsorption sites, resulting in the pre-activating of CO molecules. By tuning the side groups of NHC molecules, the CORR catalytic activity of the adatoms on Cu(100) surface can be further improved. As the electron-donating ability of the side groups increases, the adsorption interaction between CO and the adatom decreases, reducing the change in the standard Gibbs free energy for C-C coupling. These results indicate that the effectiveness of NHC decorated Cu adatoms in reducing the energy barrier for CORR, thereby providing a promising strategy for the design of efficient CORR catalysts.

## Results and discussions

NHC molecules have strong σ-donating and moderately π-accepting properties, making them particularly suitable as ligands for the transition metals, including Au, Ag and Cu^37^. Recent scanning tunneling microscopy observations and DFT calculations have uncovered various binding modes of NHCs on metallic surfaces^30,38,39^. To determine the most stable adsorption configuration, four binding modes of the NHC molecule on the Cu(100) surface were investigated, including NHC adsorption on an adatom, NHC dimers adsorption on an adatom and NHC adsorption on surface atoms in both the top and bridge sites. It is found that the NHC-adatom mode exhibits the lowest adsorption energy, suggesting that binding NHC to an adatom is favorable.

**Fig. 1:**
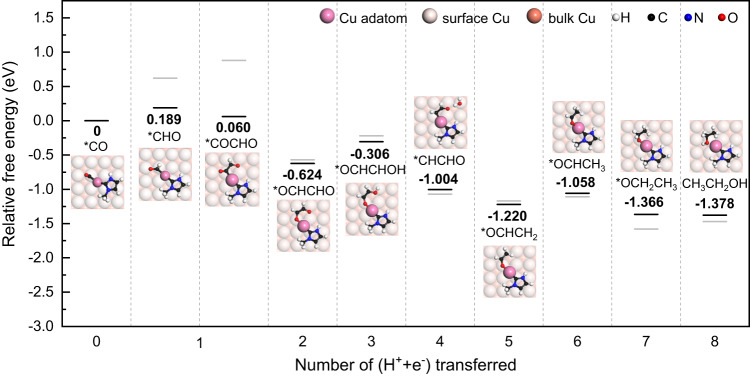
CO reduction reaction path on Cu(100) with NHC decorated adatom. The gray dashes represent the reaction path on pristine Cu(100) surface, reproduced with permission from W Luo et al.^15^.

Based on the NHC decoration, the reaction pathways of CO reduction to ethanol on Cu(100) surface with Cu adatom were investigated, as shown in Fig. 1. The black dashes represent the relative Gibbs free energies of the intermediates of each proton-electron transfer step on Cu(100) surface with NHC decorated adatom. The free energy of *CO adsorption is used as the reference. The gray dashes represent the relative Gibbs free energies of same CORR pathway on the pristine Cu(100) surface reported by previous work^15^. The results indicate that the reaction pathway for *CO reduction to ethanol on Cu(100) surface with NHC decorated adatom is similar with that on pristine Cu(100) surface, except for the first protonation of *CO and C-C coupling steps. The reaction energy for *CO hydrogenation to *CHO is 0.189 eV on the Cu(100) surface with NHC decorated adatom, much lower than that of previously reported pristine Cu(100) surface (0.62 eV), demonstrating the enhanced catalytic activity of Cu(100) surface promoted by NHC decoration. Furthermore, the reaction energy of *CO hydrogenation to *COH is 0.842 eV, 4 times that of *CHO, indicating the high selectivity for *CO hydrogenation products on Cu(100) surface with NHC decorated adatom. In contrast, the reaction energy for *COH formation on the pristine Cu(100) surface is only about 0.12 eV higher than that of *CHO. Thus, the NHC decoration improves both the reaction activity and selectivity for *CO hydrogenation to *CHO on Cu(100) surface.

The C-C coupling is essential for generating economically desirable C_2_ products. However, the coupling of *CO is impractical due to its reaction energy of 1.00 eV, while the *OCCO dimer is unstable on the Cu(100) surface^15,40^. In contrast, the coupling of *CHO and *CO is more energy-favorable on the pristine Cu(100) surface with a reaction energy of 0.26 eV^15^. After the decoration of NHC, the endothermic *CO-*CHO coupling reaction on the Cu(100) surface changes to exothermic with a reaction energy of −0.129 eV, which significantly promotes the formation of C_2_ products. Then, the *COCHO is further reduced to *OCHCHO with a reaction energy of −0.684 eV. The third protonation step at *OCHCHO favors the formation of *OCHCHOH with an uphill reaction energy of 0.318 eV. Among the subsequent steps, the reaction pathway is generally downhill towards ethanol in most cases, except for a slightly free energy increase of about 0.162 eV at the sixth protonation step.

**Fig. 2:**
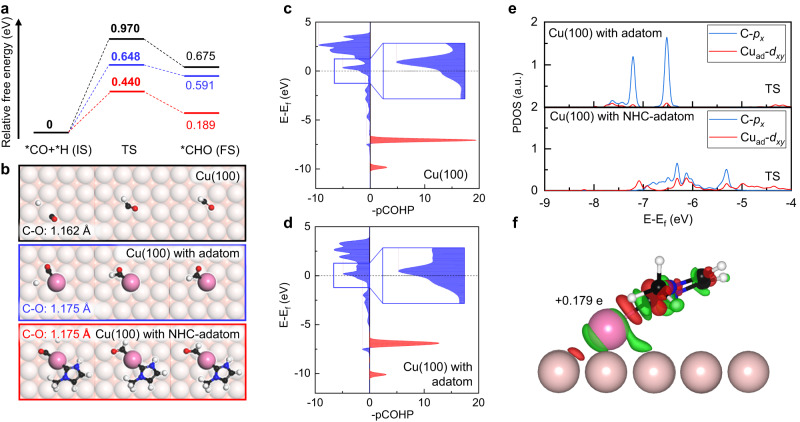
CO hydrogenation reaction on pristine Cu(100) and decorated Cu(100) surface. **a** Free energy profiles on pristine Cu(100) surface (black), Cu(100) surface with adatom (blue), and Cu(100) surface with NHC decorated adatom (red), respectively. **b** Atomic configurations of the IS, TS and FS on pristine Cu(100) surface (black), Cu(100) surface with adatom (blue), and Cu(100) surface with NHC decorated adatom (red), respectively. **c**, **d** The –pCOHP for CO adsorption on pristine Cu(100) surface and Cu(100) surface with adatom, respectively. **e** The projected density of states of the *d*_*xy*_ orbital of Cu adatom (Cu_ad_) and *p*_*x*_ orbital of C atom in TS on Cu(100) surface with and without NHC ligand, respectively. **f** The charge density difference of Cu(100) surface with NHC decorated adatom. The isosurface value is 0.001 eBohr^-3^.

To explore the origin of reaction activity improvement by NHC decoration in *CO hydrogenation reaction, the activation barriers for *CO hydrogenation are calculated on pristine Cu(100) surface, Cu(100) surface with adatom, and Cu(100) surface with NHC decorated adatom. As shown in Fig. 2a, the activation barriers for *CO hydrogenation are 0.970 eV, 0.648 eV, and 0.440 eV on the pristine Cu(100) surface, Cu(100) surface with adatom, and Cu(100) surface with NHC decorated adatom, respectively. Figure 2b shows the atomic structures of the initial state (IS), transition state (TS), and final state (FS) of the *CO hydrogenation on three surfaces according to Fig. 2a. The NHC-adatom decoration significantly reduces the activation barrier of *CO hydrogenation on the Cu(100) surface by 55%, meanwhile the adatom decoration reduces the activation barrier by 33%. This suggests that the adatom decoration makes an important contribution to the improvement of the reaction activity of Cu(100) surface. On the other hand, the configurations in Fig. 2b indicate that the adsorption configuration of *CO in IS changes after the decoration of adatoms on the surface. On the pristine Cu(100) surface, *CO is only bound to one surface atom. While on the surface with an adatom, *CO is bound to the adatom and an adjacent surface atom. The bond length of the C-O bond in IS is 1.162 Å on the pristine Cu(100) surface, while they are 1.175 Å on the Cu(100) surface with adatom and NHC-decorated adatom. The elongation of the C-O bond indicates the weakening of bond strength, which may contribute to the activation of the *CO.

To investigate the role of surface adatoms in the *CO activation, we conduct a crystal orbital Hamilton population (COHP) analysis. The projected crystal orbital Hamilton population (pCOHP) for CO adsorption on the pristine and adatom-decorated Cu(100) surfaces are presented in Fig. 2c, d, respectively. The atomic coordinates of optimized models are in Supplementary Data 1. Compared to the case of *CO adsorption on the pristine Cu(100) surface, the anti-bonding state of *CO adsorbed on the adatom-decorated Cu(100) surface is more-occupied and is at a lower energy level. The integrated overlap populations (ICOHP) up to the Fermi level of C-O bond are −16.256 and −14.675 for *CO adsorbed on the pristine and adatom-decorated Cu(100) surfaces, respectively. A higher ICOHP of *CO adsorbed on the adatom indicates a weaker C-O bond, demonstrating that the *CO is more active on the adatom site. The changes in the C-O bond suggest that the surface adatoms serve as new *CO adsorption sites, optimizing the adsorption configuration of *CO and activating it, therefore, reducing the reaction barrier of *CO hydrogenation at the first protonation step of CORR.

In addition, we further investigate the influence of NHC on the electronic structure of Cu adatom. Figure 2e presents the projected density of states (PDOS) of the *d*_*xy*_ orbital of the Cu adatom (Cu_ad_) and the *p*_*x*_ orbital of the C atom of TS during *CO hydrogenation on the Cu(100) surface with adatom and Cu(100) surface with NHC decorated adatom. The atomic coordinates of optimized models are in Supplementary Data 1. The *d*_*xy*_ orbital of the Cu adatom interacts with the *p*_*x*_ orbital of the C atom. Notably, the NHC decoration strengthens the overlap between the *d*_*xy*_ orbital of the Cu adatom and the *p*_*x*_ orbital of the C atom, leading to a much stronger interaction between the Cu adatom and *CO, resulting in an enhanced catalytic performance.

Furthermore, the charge density difference of a NHC decorated adatom is presented in Fig. 2f, revealing a significant charge transfer between the Cu adatom and the NHC molecule. The electrons accumulate between the carbene C atom and the Cu adatom, indicating the formation of a strong metal-carbene bond, while the electron depletion around the Cu adatom reveals a clear charge transfer from Cu adatom to the NHC molecule. This is also confirmed by our Bader charge analysis that the presence of NHC molecule leads to the partially oxidization of Cu adatom in a positive charge of +0.179 e. In contrast, the undecorated Cu surface adatom is uncharged. Previous studies have shown that surface Cu^δ+^ sites in Cu-based catalysts exhibit higher catalytic activity for C_2_ products compared to pure Cu, which is attributed to their enhanced adsorption strength for key reaction intermediates such as CO^41–43^. Therefore, the oxidation of Cu adatom induced by NHC decoration may contribute to a lower activation barrier, thus promoting the catalytic performance.

**Fig. 3:**
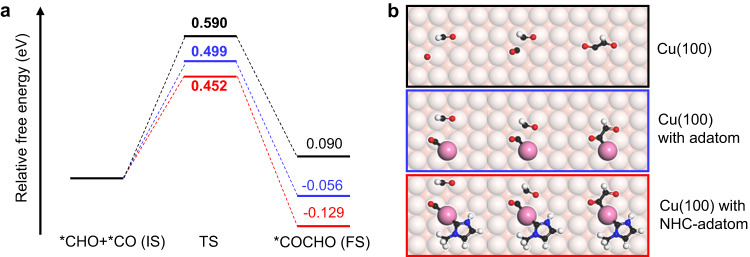
CHO-CO coupling on pristine Cu(100) and decorated Cu(100) surface. **a** Free energy profiles on pristine Cu(100) surface (black), Cu(100) surface with adatom (blue) and Cu(100) surface with NHC decorated adatom (red), respectively. **b** Atomic configurations of the IS, TS and FS on pristine Cu(100) surface (black), Cu(100) surface with adatom (blue) and Cu(100) surface with NHC decorated adatom (red), respectively.

The C-C coupling reaction is well-known to be the rate-determining step in the process of *CO reduction to C_2_ products. Thus, reducing the activation barrier of the C-C coupling is crucial for increasing the yield of C_2_ products^44^. Fig. 3a presents the activation barriers of *CO-*CHO coupling on pristine Cu(100) surface, Cu(100) surface with adatom, and Cu(100) surface with NHC decorated adatom. The activation barriers of C-C coupling are 0.590 eV, 0.499 eV and 0.452 eV on pristine Cu(100) surface, Cu(100) surface with adatom, and Cu(100) surface with NHC decorated adatom, respectively. Similar to the *CO hydrogenation process, the activation barrier of C-C coupling is reduced in the presence of surface Cu adatom and further decreases through NHC decoration.

The atomic structures of the IS, TS and FS of C-C coupling on three surfaces are shown in Fig. 3b, which reveals that the adsorption configuration of *COCHO undergoes significantly changes after the introduction of Cu adatom. On the pristine Cu(100) surface, the bond order of C-O in *O-CCHO is 1.34, which is close to that of a C-O single bond. The bond order of the C-C is 1.31, which is close to that of a C-C single bond. While on the Cu(100) with adatom and NHC decorated adatom, the bond orders of C-O in *O-CCHO are 1.91 and 1.86, resembling C = O double bonds. The bond orders of C-C are 1.16 and 1.15, resembling C-C single bonds. These changes in bond order indicate the enhanced stability of *COCHO on the surface adatom compared to the pristine Cu(100) surface. This result shows that the presence of surface adatom stabilizes *COCHO, which reduces the Gibbs free energy of the FS. Thus, similar to the step of *CO hydrogenation, the atom formed by NHC adsorption on Cu(100) surface stabilize the crucial intermediate (*COCHO) and driving the C-C coupling from endothermic to exothermic.

**Fig. 4:**
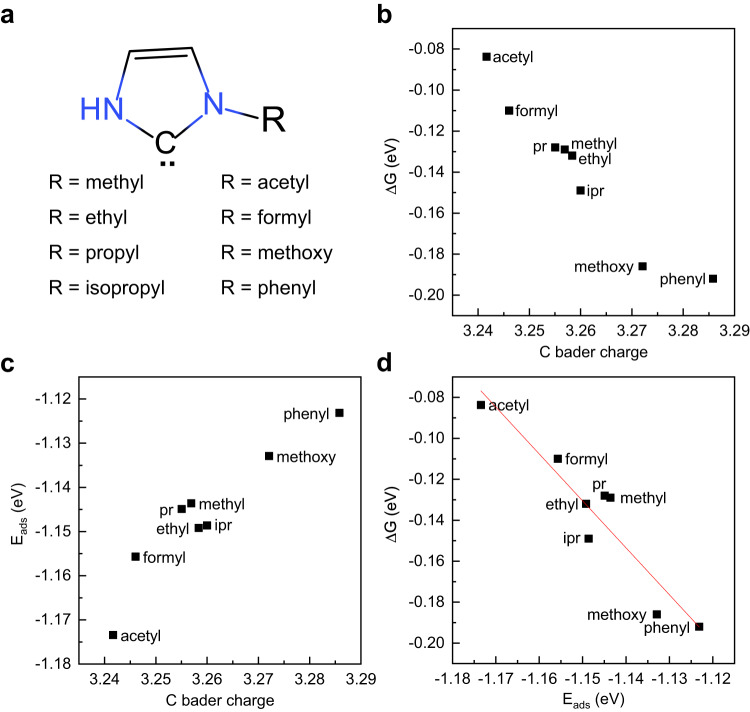
Effect of side group substitutions on CHO-CO coupling. **a** Structural formulas of NHC molecules with side groups studied. **b** The dependence between ΔG of CHO-CO coupling and the Bader charge of carbene C atom. **c** The dependence between adsorption energies of CO (E_ads_) and the Bader charge of carbene C atom. **d** A scaling relation between ΔG and E_ads_.

To investigate the influence of molecular electronic properties of NHC on the Cu(100) surface with NHC decorated adatom and further improve its catalytic activity, the σ-donation and π-acceptor ability of NHC molecules is adjusted by tuning the side groups. Therefore, 8 kinds of side groups are considered, including methyl, ethyl, propyl, isopropyl, acetyl, formyl, methoxy and phenyl, in a range of electronic properties from electron-donating to electron-withdrawing, as shown in Fig. 4a. Among them, methyl, ethyl, propyl and isopropyl are weak electron-donating groups, acetyl and carboxyl are strong electron-withdrawing groups, while methoxy and phenyl are strong electron-donating groups.

To assess the effect of side group substitution on the activation barriers, the Bader charge of the carbene C atom and the change in standard Gibbs free energy (ΔG) of C-C coupling are calculated to provide insights into the NHC-decorated system. According to the Brønsted-Evans-Polanyi relationship, there is a linear correspondence between the activation barrier and the ΔG in surface reactions^45,46^. As shown in Fig. 4b, the ΔG of C-C coupling decreases with the increase of the Bader charge of the carbene C atom. It indicates that the catalytic activity of Cu adatoms decorated by NHC molecules is enhanced by the strong electron-donating side group. Furthermore, the adsorption energies of the reactant *CO on the Cu adatoms decorated by NHC with different side groups are calculated. Figure 4c illustrates that the adsorption energies of *CO (E_ads_) increase as the Bader charges on the carbene C atoms increase, indicating that the stronger the electron-donating ability of the side groups results in weaker adsorption of *CO on the NHC-decorated Cu adatoms. A scaling relation between reaction energies and *CO adsorption energies is in Fig. 4d, similar to the previous works^47^. Thus, the reduction of reaction energy can be attributed to the increase in *CO adsorption energy, which is highly correlated to the electronic structure of Cu adatom adjusted by side group substitution.

## Conclusions

In conclusion, our DFT calculations predict that NHC decoration is a promising strategy to improve the catalytic activity of Cu adatoms on Cu(100) surfaces for CORR. The relative Gibbs free energy diagrams indicate that the decoration of NHC molecules significantly reduces both the standard Gibbs free energy change and the activation barrier of CO hydrogenation and the C-C coupling on the Cu(100) surface with Cu adatom. The reduction in reaction barrier for CORR is mainly attributed to the enhanced interaction between the *d*_*xy*_ orbital of Cu adatoms and the *p*_*x*_ orbital of C atom in CO, facilitated by the NHC decoration. Furthermore, the catalytic activity of Cu adatom can be tailored by varying the side group of NHC molecules. Our DFT calculations reveal that the side groups with strong electron-donating ability result in weak *CO adsorption, leading to the low reaction energies of C-C coupling. This finding provides a considerably practical strategy for the design active catalysts for CORR.

## Methods

First-principle calculations based on DFT were performed by using the Vienna ab initio simulation package^48,49^. The projector augmented wave^50^ method was applied with Perdew-Burke-Ernzerhof^51^ type exchange-correlation functional. Dispersion correction was included using the DFT-D3 method of Grimme^52^. A kinetic energy cutoff of 400 eV was applied for all calculations. The k-points sampling was 2 × 2 × 1, generated by Monkhorst–Pack grids with the origin at the Γ-point^53^. The convergence criterion of electronic relaxation was 10^−6^ eV.

The Cu(100) slab model was constructed with four atomic layers and a 6 × 6 supercell containing a total of 144 Cu atoms. A vacuum layer of 20 Å was placed above the slabs to avoid interactions between the periodic images. All atoms were fully relaxed except the two bottom layers of Cu. The structures were relaxed until the residual force on each atom was smaller than 0.01 eVÅ^-1^.

Free energies, G, were computed with the energetic correction terms according to the following equation:

$$G={E}_{{electronic}}+{ZPE}+\int {C}_{p}{dT}-{TS}$$

where the ${E}_{{electronic}}$ was obtained by DFT calculations, the entropy ($S$), the zero-point energies (${ZPE}$) of adsorbates and the heat capacity (${C}_{p}$) were derived from the vibrational frequency calculations^10,54^. The temperature ($T$) were set to be at 300 K. The anharmonic terms were not included. In the case of adsorbed molecules, the contributions from translation and rotation to entropy are significantly reduced and turn into vibrational modes^55^. For the vibrational frequency calculations, the convergence criterion of electronic relaxation was 10^−7^ eV. The computational hydrogen electrode (CHE) approach was used for proton−electron transfer steps^56^. Transition states were calculated using the climbing image nudged elastic band^57,58^ and dimer methods^59^ for CO hydrogenation and C-C coupling.

### Supplementary information

Supplementary Data 1

## Data Availability

The authors declare that data supporting the findings of this study are available within the paper. The atomic coordinates of the optimized computational models for electronic structure calculations are in Supplementary Data 1. Additional data are available from the corresponding author on reasonable request.
